# Baicalein Ameliorates Myocardial Ischemia Through Reduction of Oxidative Stress, Inflammation and Apoptosis via TLR4/MyD88/MAPK_S_/NF-κB Pathway and Regulation of Ca^2+^ Homeostasis by L-type Ca^2+^ Channels

**DOI:** 10.3389/fphar.2022.842723

**Published:** 2022-03-16

**Authors:** Jinghan Li, Yakun Yang, Hua Wang, Donglai Ma, Hongfang Wang, Li Chu, Yuanyuan Zhang, Yonggang Gao

**Affiliations:** ^1^ School of Basic Medicine, Hebei University of Chinese Medicine, Shijiazhuang, China; ^2^ School of Pharmacy, Hebei University of Chinese Medicine, Shijiazhuang, China; ^3^ Hebei Key Laboratory of Integrative Medicine on Liver-Kidney Patterns, Hebei University of Chinese Medicine, Shijiazhuang, China

**Keywords:** baicalein, myocardial ischemia, TLR4/MyD88/MAPKs/NF-κB pathway, l-type Ca2+ channels, inflammation, apoptosis

## Abstract

**Background:** Baicalein (Bai) is the principal ingredient of *Scutellaria baicalensis Georgi*. Reports concerning the therapeutic advantages in treating cardiovascular diseases have been published. However, its protective mechanism towards myocardial ischemia (MI) is undefined.

**Objective:** The aim of this study was to investigate the protective mechanisms of Bai on mouse and rat models of MI.

**Methods:** Mice were pre-treated with Bai (30 and 60 mg/kg/day) for 7 days followed by subcutaneous injections of isoproterenol (ISO, 85 mg/kg/day) for 2 days to establish the MI model. Electrocardiograms were recorded and serum was used to detect creatine kinase (CK), lactate dehydrogenase (LDH), superoxide dismutase (SOD), catalase (CAT), glutathione (GSH) and malondialdehyde (MDA). Cardiac tissues were used to detect Ca^2+^ concentration, morphological pathologies, reactive oxygen species (ROS), interleukin-6 (IL-6) and tumor necrosis factor-α (TNF-α). In addition, the expression levels of Bcl-2-associated X (Bax), B cell lymphoma-2 (Bcl-2), Caspase-3, Toll-like receptor-4 (TLR4), myeloid differentiation protein 88 (MyD88), nuclear factor-kappa B (NF-κB), p-p38, p-extracellular signal-regulated kinase1/2 (*p*-ERK1/2) and c-Jun N-terminal kinase (*p*-JNK) were assessed by western blots in myocardial tissues. The effects of Bai on L-type Ca^2+^ currents (I_Ca-L_), contractility and Ca^2+^ transients in rat isolated cardiomyocytes were monitored by using patch clamp technique and IonOptix system. Moreover, ISO-induced H9c2 myocardial injury was used to detect levels of inflammation and apoptosis.

**Results:** Bai caused an improvement in heart rate, ST-segment and heart coefficients. Moreover, Bai led to a reduction in CK, LDH and Ca^2+^ concentrations and improved morphological pathologies. Bai inhibited ROS generation and reinstated SOD, CAT and GSH activities in addition to inhibition of replenishing MDA content. Also, expressions of IL-6 and TNF-α in addition to Bax and Caspase-3 were suppressed, while Bcl-2 expression was upregulated. Bai inhibited protein expressions of TLR4/MyD88/MAPK_S_/NF-κB and significantly inhibited I_Ca-L_, myocyte contraction and Ca^2+^ transients. Furthermore, Bai caused a reduction in inflammation and apoptosis in H9c2 cells.

**Conclusions:** Bai demonstrated ameliorative actions towards MI, which might have been related to attenuation of oxidative stress, inflammation and apoptosis via suppression of TLR4/MyD88/MAPK_S_/NF-κB pathway and adjustment of Ca^2+^ homeostasis via L-type Ca^2+^ channels.

## 1 Introduction

Ischemic heart disease is the leading cause of disease burden worldwide (Lopez and Murray, 1998). Cardiomyocyte death resulting from myocardial ischemia (MI) underlies the most significant cardiovascular-related deaths (Cannon, 2005). MI usually happens in the middle-aged and elderly population, however, with the increasing competition in society and the increasing pressure on young people, its onset tends to be younger and younger (Ingram et al., 2013). Therefore, the prevention and treatment of myocardial ischemia have significant implications in the future.

MI results in a reduction in the supply of oxygen and abnormal energy metabolism that is inadequate to ensure healthy heart function (Hausenloy and Yellon, 2013). Long-lasting ischemia causes irreversible cellular damage and eventually apoptosis. Inflammation and oxidative stress are both closely associated with MI (Matin et al., 2020; Mo et al., 2021). Pro-inflammatory cytokines exert a crucial role in heart injury (Lauer et al., 2014), and the Toll-like receptor-4/nuclear factor-kappa B (TLR4/NF-κB) pathway regulates the secretion of these cytokines. Myeloid differentiation protein 88 (MyD88) is an adapter protein that is critical for TLR4 (Singh et al., 2012) and causes enhancement of inflammation and an increase in reactive oxygen species (ROS) production (W. Gao et al., 2016). After the cardiac injury, NF-κB activates and evokes transcription of pro-inflammatory cytokines (Wu et al., 2018; Han et al., 2020). Therefore, inhibition of the TLR4/NF-κB pathway averts inflammation (Hirotani et al., 2002; Lawrence, 2009; X.; Wang, 2001). NF-κB can activate mitogen-activated protein kinases (MAPKs), MAPKs are important for regulating apoptosis (Du et al., 2017).

Calcium ions are important in many aspects of normal cardiac function as well as in the response to certain pathologic states. Enhanced contractility of cardiomyocytes is a central characteristic of cardiac response in ischemic cardiomyopathy (Bristow et al., 1985; Y.; Gao et al., 2014). Ca^2+^ enters cardiomyocytes principally via L-type Ca^2+^ channels (LTCC) as described by several groups of researchers (Piper et al., 1998; Ferrier and Howlett, 2001). The increase in intracellular Ca^2+^ leads to enhanced myocardial contractility and accelerated pathological changes (Frey and Olson, 2003; Chen et al., 2005). Calcium channel blockers are capable of alleviating MI by inhibiting LTCCs, interfering with Ca^2+^ influx and weakening myocardial contractility. Our previous studies also have demonstrated that drugs specifically attenuating L-type Ca^2+^ currents are promising agents concerning exerting cardiac protection (Song et al., 2016; Han et al., 2019).

**FIGURE 1:**
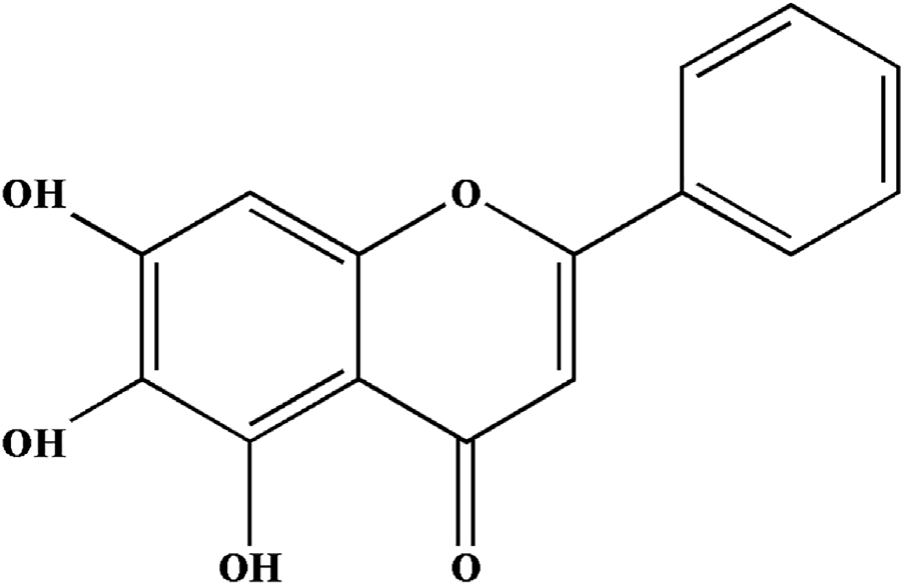
Chemical structure of Baicalein (Bai).

*Scutellaria baicalensis* is a traditional Chinese medicine with a variety of pharmacological functions for treating cardiovascular disease and hypertension (Li-Weber, 2009). Baicalein (5, 6, 7-Trihydroxyflavone, C_15_H_10_O_5_, Bai, Figure 1) is the main active flavonoid constituent in *S. baicalensis Georgi* plants (Xiping et al., 2007; Dinda et al., 2017). Bai is an antioxidant (Kim et al., 2018) and has potent anti-inflammatory activities (Luo et al., 2017). Recently, emerging evidence demonstrated that Bai could suppress apoptosis in response to simulated ischemia/reperfusion (L. Song et al., 2014). Nevertheless, the mechanism of Bai against MI has not been reported.

Given the above-mentioned facts, we intended to evaluate the mechanisms underlying the cardioprotective actions of Bai against ISO-induced MI based on the inhibition of the TLR4/MyD88/MAPK_S_/NF-κB signaling pathway and the regulation of Ca^2+^ homeostasis. This study would not only assist in better elaborating the role of Bai in clinical treatment but would also provide an experimental basis for the reasonable application of Bai.

## 2 Materials and Methods

### 2.1 Drugs and Reagents

Bai (purity >99%) was obtained from Damas-Beta (Shanghai Titan Scientific Co., Ltd), ISO was purchased from Amylet Scientific Inc. (Michigan, United States), and verapamil (Ver) was provided by Harvest Pharmaceutical Co., Ltd. (Shanghai, China). Nicardipine (Nic) was obtained from Sigma Chemical Co. (St. Louis, Mo). Bai was diluted into the proper concentrations in carboxymethyl cellulose and used immediately. ISO and Ver were dissolved in normal saline. Dulbecco’s modified Eagle’s medium (DMEM, Catalog: 12430047), fetal bovine serum (Catalog: 10093188), trypsin (Catalog: 25200072), and penicillin/streptomycin were purchased from Gibco (Thermo Fisher Scientific, Inc. Missouri, United States). Unless otherwise specified, other laboratory chemicals were obtained from Sigma-Aldrich (St. Louis, MO, United States) and were of analytical purity.

### 2.2 Animals

Forty Kunming male mice (20 ± 2 g; 4–5 weeks) and male adult Sprague-Dawley rats (180–220 g; 42–45 days) were obtained from the Experimental Animal Centre, Hebei Medical University. Before the experiments, animals were brought into the laboratory and allowed to acclimate for 1 week. Mice were caged in 22 ± 2°C with 12 h light/dark cycles and supplied with adequate food and water. The experiments were performed according to the International Association of Veterinary Editors guidelines and approved by the Ethics Committee for Animal Experiments of Hebei University of Chinese Medicine (approval number: DWLL2020073).

### 2.3 Experimental Design

Forty male Kunming mice were randomly allocated into five groups with eight mice per group. The doses of ISO and Bai are can be obtained from previous references (Haleagrahara et al., 2011; Dinda et al., 2017). Details of the experimental groups are as follows:

Control (Con): Mice were intraperitoneally injected (i.p.) with 0.9% normal saline (0.1 M/kg/day) once each day for 7 days.

ISO: Mice were subcutaneously administered ISO (85 mg/kg, s. c.) on the last two consecutive days of the experiment.

Low-dose Bai (L-Bai): Mice in the L-Bai group were pre-treated with Bai solution (30 mg/kg/day) via gavage for 7 days. Then mice were subcutaneously administered ISO (85 mg/kg, s. c.) on the last two consecutive days of the experiment.

High-dose Bai (H-Bai): Mice were pre-treated with Bai solution (60 mg/kg/day) via gavage for 7 days. Then mice were subcutaneously administered ISO, the same as the L-Bai group.

Ver: Mice were given Ver (2 mg/kg/day, i. p.) for 7 days. Then ISO was subcutaneously administered, the same as L-Bai and H-Bai groups.

Twenty-four h after the last treatment, pentobarbital sodium (50 mg/kg) was injected i. p. to anesthetize the mice (B. Zheng et al., 2021). The electrocardiogram (ECG) was recorded on anesthetized mice. After that, blood was collected and serum was separated by centrifugation. Then hearts were quickly dissected, rinsed with saline, weighed, and photographed. For further detection, the heart tissues were placed into liquid nitrogen.

### 2.4 Determination of Electrocardiogram

After anesthesia, the RM6240BD Biological Signal Collection System (Chengdu Manufacture Factory) was used to record the ECG and monitor changes in heart rate and ST-segment elevation in each group of mice. ECG was recorded using three subcutaneous needle electrodes: 1) green electrode connected to the right upper limb; 2) black electrode connected to the right lower limb; and 3) red electrode connected to the left lower limb.

### 2.5 Estimations of Cardiac Marker Enzymes

Mice were anesthetized, blood was drawn from the enucleation of the eyeball and collected in heparinized tubes. The serum was separated by centrifugation at 3,500 rpm for 10 min. Creatine serum (CK) in serum (No. A032-1-1) and lactate dehydrogenase (LDH) levels (No. A020-2-2) were measured to estimate myocardial damage via a colorimetric method using standard diagnostic kits (Jian Cheng Biological Engineering Institute, Nanjing, China). A UNICO-UV2000 spectrophotometer (JianCheng, Nanjing, China) was used to measure corresponding absorbances.

### 2.6 Detections of Ca2+ Concentration

The concentration of Ca^2+^ (No. C004-2-1) was assessed in the myocardium. Heart tissue was homogenized in 10% deionized water. The homogenate was centrifuged at 2,500 rpm for 10 min and the supernatant was used for the assay. After the samples were processed, the BCA kit was used to detect the protein concentration. The concentration of Ca^2+^ was measured by methylthymol blue staining following the commercially available kit’s instructions (Jiancheng, Nanjing, China). Finally, the OD value of each well was measured at 610 nm using an enzyme marker.

### 2.7 Histopathology Observations

The heart samples were prepared and immediately stocked in 4% paraformaldehyde. After 24 h, samples were routinely processed and embedded in paraffin. Samples were sectioned (5 μm) and stained using hematoxylin and eosin (H&E). Sections were visualized via light microscopy (Leica DM4000B, Solms, Germany).

### 2.8 Oxidative Stress Detection

#### 2.8.1 Probing for ROS Production

The fluorescent probe, dihydroethidium (DHE, Cat. Beyotime Institute of Biotechnology, Shanghai, China), was used to measure the amount of ROS in heart tissues. Heart tissues were added to an optimum cutting temperature (OCT) embedding medium (No. 4583; Sakura) and then frozen in dry ice. Frozen slides were brought to room temperature, and excess liquid was discarded. The target tissue was marked with a fluid blocker pen. ROS staining solution (No. G1045; Servicebio, Wuhan, China) was added to the marked area, and the slide was incubated at 37°C and kept in the dark for 30 min. After washing three times with phosphate-buffered saline (PBS) at pH 7.4 (No. G002; Servicebio, Wuhan, China) in a Rocker device for 5 min each, 4’,6-diaminidino-2-phenylindole (DAPI) solution was dripped onto the slide (No. G1012; Servicebio, Wuhan, China) and incubated at room temperature in the dark. Ten minutes later, the slices were washed using the previous method and sealed with an anti-fade mounting medium coverslip (No. G1401; Servicebio, Wuhan, China). Images of ROS generation were collected using fluorescence microscopy. Red fluorescence labeling was detected when ROS was generated and quantified using Image-Pro Plus software.

#### 2.8.2 SOD, CAT, GSH, and MDA Detection

The serum samples were checked for the activities of superoxide dismutase (SOD, No. A001-3-1), and catalase (CAT, No. A007-1-1), glutathione (GSH, No. A006-2-1) and the level of malondialdehyde (MDA, No. A003-1-1). These antioxidant enzymes and lipid peroxides were detected by spectrophotometry using commercially available kits (Jiancheng, Nanjing, China).

### 2.9 Measurements of Interleukin-6 (IL-6) and Tumor Necrosis Factor-α (TNF-α)

IL-6 (No. 88-7064) and TNF-α (No. 88-7324) expressions in heart tissues were measured to test the inflammatory status in mice of the five groups using enzyme-linked immunosorbent assay (ELISA) kits according to manufacturer’s instructions (Servicebio, Wuhan, China).

### 2.10 Assessment of Bcl-2-Associated X (Bax), B Cell Lymphoma-2 (Bcl-2), Caspase-3, and TLR4/MyD88/NF-κB/MAPKs Pathway by Western Blot

As previously described (Zhang et al., 2016), the cardiac tissues were dissolved in RIPA lysis buffer (No. G2002; Servicebio, Wuhan, China). The supernatant containing total protein extract was used to determine the concentration using the BCA protein quantitative assay kit (No. G2026; Servicebio, Wuhan, China) after centrifugation (12,000 rpm, 10 min, 4°C). Heart tissue lysates were loaded onto a 10% SDS-PAGE gel (No. G2003; Servicebio, Wuhan, China), transferred into polyvinylidene fluoride membranes (No. G6015–0.45; Servicebio, Wuhan, China), and blocked with TBST buffer carrying 5% (w/v) skim milk at 37°C for 30 min. The membranes underwent overnight incubation at 4°C with primary antibodies against Bax (No. GB11690; Servicebio, Wuhan, China; diluted 1:1,000), Bcl-2 (No. PAA778Mu01; Cloud-Clone, Wuhan, China; diluted 1:1,000), Caspase-3 (No. 66470-2-lg; San Ying, Wuhan, China; diluted 1:1,000), TLR4 (No. GB11519; Servicebio, Wuhan, China; diluted 1:1,000), MyD88 (No. 23230-1-AP; San Ying, Wuhan, China; diluted 1:1,000), polyclonal anti-NF-κB (p65) (No. GB11142; Servicebio, Wuhan, China; diluted 1:1,000), p-p38 (no. 4511; Cell Signaling Technology, Massachusetts, United States; diluted 1:1,000), p-extracellular signal-regulated kinase1/2 (*p*-ERK1/2, No. 4370; Cell Signaling Technology, Massachusetts, United States; diluted 1:1,000), and c-Jun N-terminal kinase (*p*-JNK, no. 4668; Cell Signaling Technology, Massachusetts, United States; diluted 1:1,000). Anti-β-actin (No. GB12001; Servicebio, Wuhan, China; diluted 1:1,000) was used as the internal standard. The next day, the membrane was washed with PBS-T (No. G0001-2L; Servicebio, Wuhan, China) and incubated with horseradish peroxidase (HRP)-conjugated secondary anti-rabbit or anti-mouse antibodies (No. GB23303 or No. GB23301; Servicebio, Wuhan, China; diluted 1:3,000) for 30 min at 37°C. The bound antibodies were visualized using the enhanced chemiluminescence (ECL) system (No. G2014; Servicebio, Wuhan, China) and quantified by densitometry using an Alpha Ease FC system (Alpha Innotech, Shanghai, China).

### 2.11 Isolation of Rat Ventricular Myocardium

Single ventricular myocardia were isolated from normal experimental rats that were anesthetized by injection of i. p. heparin sodium (500 IU/kg) and ethyl carbamate (1.0 g/kg). The hearts were promptly dissected, soaked in frozen Tyrode’s solution, and suspended in Langendorff equipment perfused by oxygenated frozen free Ca^2+^ Tyrode’s solution for 5 min. After that, an enzymatic solution (Collagenase Type Ⅱ, ThermoFisher, No. 17101-015) was applied to digest the heart for 15–20 min. Subsequently, the heart was removed, and the tissue was cleaned thoroughly with Tyrode’s solution. The ventricle was torn into tiny pieces in Kreb’s buffer solution. The freshly dissociated cardiomyocytes were preserved in Kreb’s buffer solution filled with oxygen for up to 1 h at room temperature before subsequent experiments were performed. Solutions used in the perfusion process must be oxygenated with 100% O_2_ and prepared as described in Table 1. The pH of normal Tyrode’s solution and the enzyme solution was adjusted to 7.4 using 3 M NaOH. The external/internal solution was adjusted to pH 7.3 with CsOH, and the Kreb’s buffer solution was adjusted to pH 7.2 using KOH.

**TABLE 1:** Components of solutions used in the study.

Solution components (unit in mM)	Normal Tyrode’s solution	The enzyme solution	The external solution	The internal solution	Kreb’s buffer
Hepes free acid (HEPES)	10	10	10	10	10
Glucose	10	10	10	—	10
CaCl_2_	1.8	0.03	1.8	—	
MgCl_2_	1	1	2	—	—
Taurine	10	4.4	—	—	20
NaCl	135	135	—	—	—
KCl	5.4	5.4	—	—	—
NaH_2_PO_4_	0.33	0.33	—	—	—
Bovine serum albumin	—	0.5^a^	—	—	—
Collagenase type Ⅱ	—	0.6^a^	—	—	—
Tetraethylammonium chloride (TEA-Cl)	—	—	140	20	—
CsCl	—	—	—	120	—
Mg-ATP	—	—	—	5	—
EGTA	—	—	—	10	1
MgSO_4_	—	—	—	—	3
KCl	—	—	—	—	40
KH_2_PO_4_	—	—	—	—	25
KOH	—	—	—	—	80
Glutamic acid	—	—	—	—	50

a Unit in mg/mL.

Myocardial ischemia was induced in the rats by subcutaneous injection with ISO (85 mg/kg). The dose and pattern of injection were carried out according to a previous study (Han et al., 2019). After two consecutive days of ischemia were established, and experimental treatments were performed as described above to isolate ventricular cells from normal rats.

### 2.12 Electrophysiological Recordings of L-type Ca2+ Currents

Whole-cell patch clamp recordings were performed in ventricular myocytes to measure the L-type Ca^2+^ currents. The pipette puller (Sutter Instrument, Novato, CA, United States) was employed for pulling the borosilicate glass microelectrodes of 3–5 MΩ resistance, which were later filled with a pipetted internal solution. I_Ca-L_ was recorded using an Axon Patch 200B amplifier with a 2 kHz applied to recordings. Data analyses were done using the Pclamp10.0 software (Axon Instruments, Union City, CA, United States). All experiments were performed in an air-conditioned room (23–25°C).

### 2.13 Measurements of Myocyte Contractions and Ca2+ Transients

Ventricular myocyte contractions and Ca^2+^ transients were detected using the IonOptix detection system (Ion Optix, Milton, Massachusetts, United States). Cardiac myocytes placed in the bath were perfused with normal Tyrode’s solution. Myocardial shortening was initiated twice by field stimulation (0.5-Hz frequency, 2-ms duration) and observed under inverted microscopy. Only cardiomyocytes with a distinct vein were suitable for measuring contractions. Cardiomyocytes were dyed in the dark for 15 min with the fluorescent indicator fura-2/AM (1 mmol/L) and illuminated by alternating between 340 and 380 nm filters. The emitted fluorescence was tested at 510 nm, and Ca^2+^ transients were recorded with the Ion Optix (United States) fluorescence system.

### 2.14 Cell Culture and Treatment

Rat H9c2 cardiomyocytes were purchased from Bluefbio (Shanghai, China, No. BFN60804388) and cultured in high-glucose DMEM which contained 10% fetal bovine serum (Gibco, Grand Island, NY, United States, No. 2176377) and 100 U/mL penicillin/streptomycin under 5% CO_2_ and 95% air at 37°C. The cell culture reagents were changed once every 2 days. H9c2 cardiomyocytes were digested with 0.25 g/L trypsin (Gibco) at approximately 80% cell confluence to be used for subculturing (Y. Zheng et al., 2019). The cells were seeded in 25-cm^2^ culture flasks and passaged every 3 days. The follow-up experiments were performed after H9c2 cardiomyocytes had reached the logarithmic growth phase. H9c2 cardiomyocytes were divided into four groups (A) Con group; (B) ISO group at 50 μmol/L (Hu et al., 2013; Lin et al., 2017); (C) L-Bai group (10 μmol/L); (D) H-Bai group (30 μmol/L).

### 2.15 Measurements of Intracellular IL-6 and TNF-α by ELISA

After the experiment, H9c2 cardiomyocytes were digested with a moderate amount of trypsin and centrifuged for 10 min at 1,500 rpm. The supernatant was discarded, and 500 μL phosphate-buffered saline (PBS) was added to resuspend the cell precipitate after which it was the mix. After that, cells were ruptured 10 times via ultrasound for 2 s to collect cell homogenate for detection. The concentration of IL-6 (No. H007) and TNF-α (No. H052) were determined by enzyme-linked immunosorbent (ELISA) kits according to the manufacturer’s instructions.

### 2.16 Assessment of Apoptosis by Hoechst 33258 Staining

As a nucleus-specific dye, Hoechst 33258 (Beijing Solarbio Science & Technology, Beijing, China, No. IH0060) is used to evaluate the extent of apoptosis. Following the experiment, the cell culture media in 24-well plates from different groups were discarded, and H9c2 cardiomyocytes were washed three times with PBS (No. 003,002). After washing, cells in each group were incubated in 500 μL/well of Hoechst 33258 staining solution (10 μg/ml) for 20 min in the dark at room temperature, and they were then rinsed twice with PBS. The cells were observed and photographed immediately under fluorescence microscopy (Leica, Germany) (Y. Zheng et al., 2019). Nuclei with brightly stained and bright fragments were regarded as apoptotic cells, while cells with blue chromatin and organized structure were considered to be normal.

### 2.17 Data Analysis

The resulting data are shown as mean ± standard error of the mean (S.E.M.) Statistical analyses were performed using Origin Pro version 9.1 software. One-way analysis of variance (ANOVA) followed by Tukey’s *post hoc* tests was performed to compare more than two groups. A value of *p <* 0.05 was considered statistically significant.

## 3 Results

### 3.1 Effects of Bai on ECG

**FIGURE 2:**
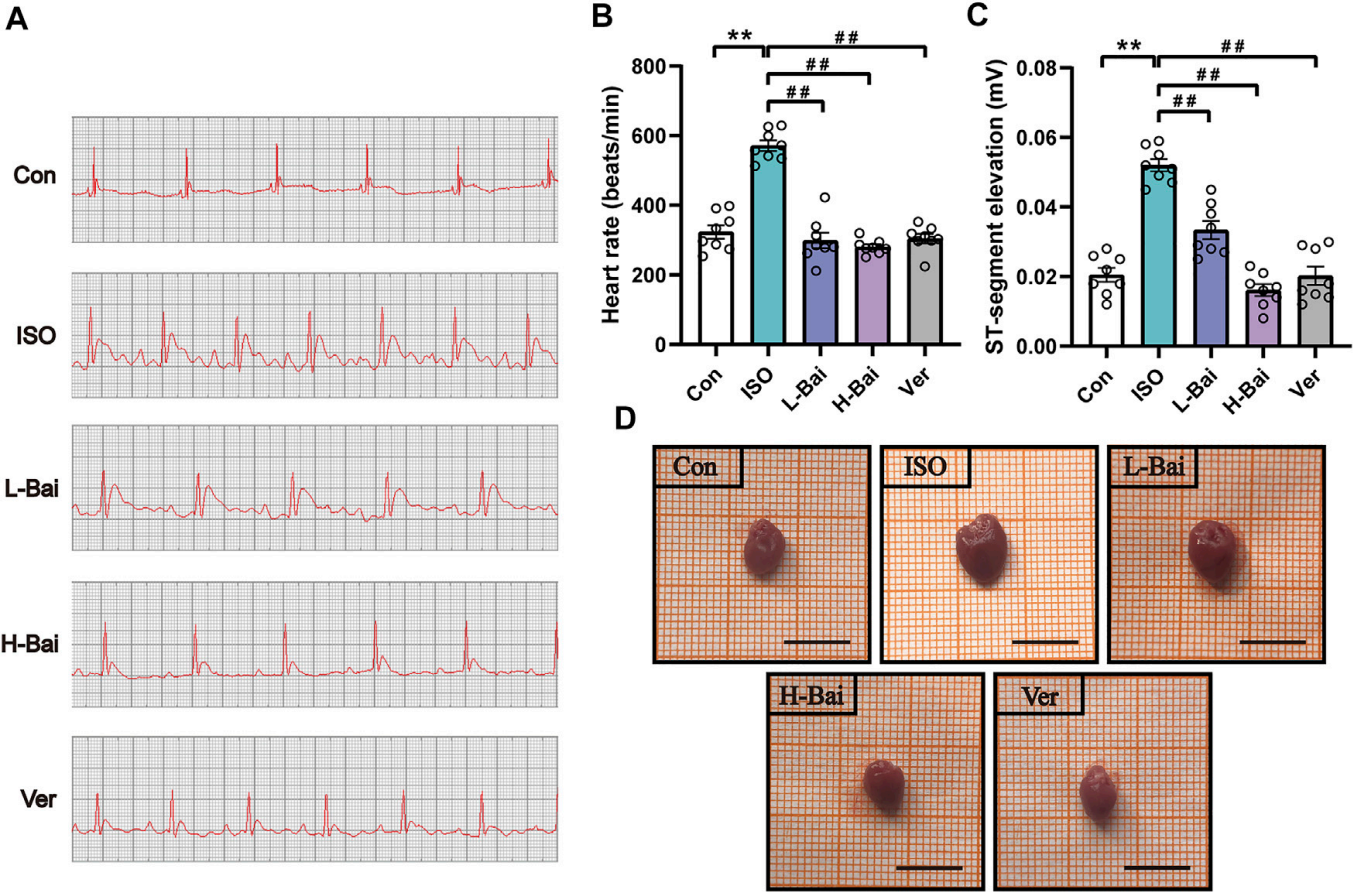
**(A)** Representative electrocardiogram (ECG) tracings of each group in mice. **(B)** Statistical analysis of heart rate **(C)** ST segment in each group. **(D)** Effects of Bai on heart appearance in mice. Scale bar = 1 cm. Data are the means ± standard error of the mean (S.E.M.) for n = 8. *p <* 0.01 versus control (Con);*p <* 0.01, *p <* 0.05 versus isoproterenol (ISO).

Figure 2A shows representative ECG tracings. As shown in Figures 2B,C, the heart rate and ST-segment showed significant elevations in ISO-induced MI mice (*p <* 0.01). Heart rate and ST-segment in L-Bai, H-Bai, and Ver groups had decreased when compared with ISO group (*p <* 0.01 or *p <* 0.05). The results revealed that Bai inhibited heart rate and ST segment in a dose-dependent manner in mice.

### 3.2 Effects of Bai on Heart Appearance, Coefficients

As shown in Figure 2D and Table 2, a marked difference between the Con and ISO groups in terms of gross appearances and body and heart weights was found. Compared to other groups, ISO treatment caused an enlargement of the heart and a significant decrease in body weight in mice, while heart weights from mice with cardiac hypertrophy increased. At the same time, the heart coefficient in ISO-treated hearts was markedly elevated (*p <* 0.01 or *p <* 0.05). In contrast, L-Bai, H-Bai, and Ver administrations resulted in a significant improvement of pathology in addition to the reduction in heart weight, heart coefficients, and augmentation in body weight (*p <* 0.01 or *p <* 0.05). These findings suggested that Bai improved the gross appearance and coefficients of the heart in a dose-dependent manner.

**TABLE 2:** Effects of Bai on heart coefficients in mice.

Groups	HW (mg)	BW (g)	Heart coefficients (mg/g)
Con	111.00 ± 7.7	33.375 ± 0.436	3.31491 ± 0.4263
ISO	143.05 ± 3.25^**^	26.067 ± 0.491^**^	5.56207 ± 0.2666^**^
L-Bai	127.12 ± 1.81^#^	26.300 ± 0.966	4.76463 ± 0.1803^#^
H-Bai	124.12 ± 3.38^##^	30.880 ± 0.762^##^	4.01981 ± 0.0558^##^
Ver	124.62 ± 1.27^##^	33.367 ± 0.606^##^	3.83765 ± 0.2523^##^

BW, body weight; HW, heart weight; heart coefficients, heart weight (HW) to-body weight (BW) ratio. Data are presented as mean ± S.E.M. (n = 8). Compared to the Con group (*p* < 0.01); compared to the ISO, group (*p* < 0.01, *p* < 0.05).

### 3.3 Effects of Bai on Cardiac Marker Enzymes and Ca2+ Concentration

**FIGURE 3:**
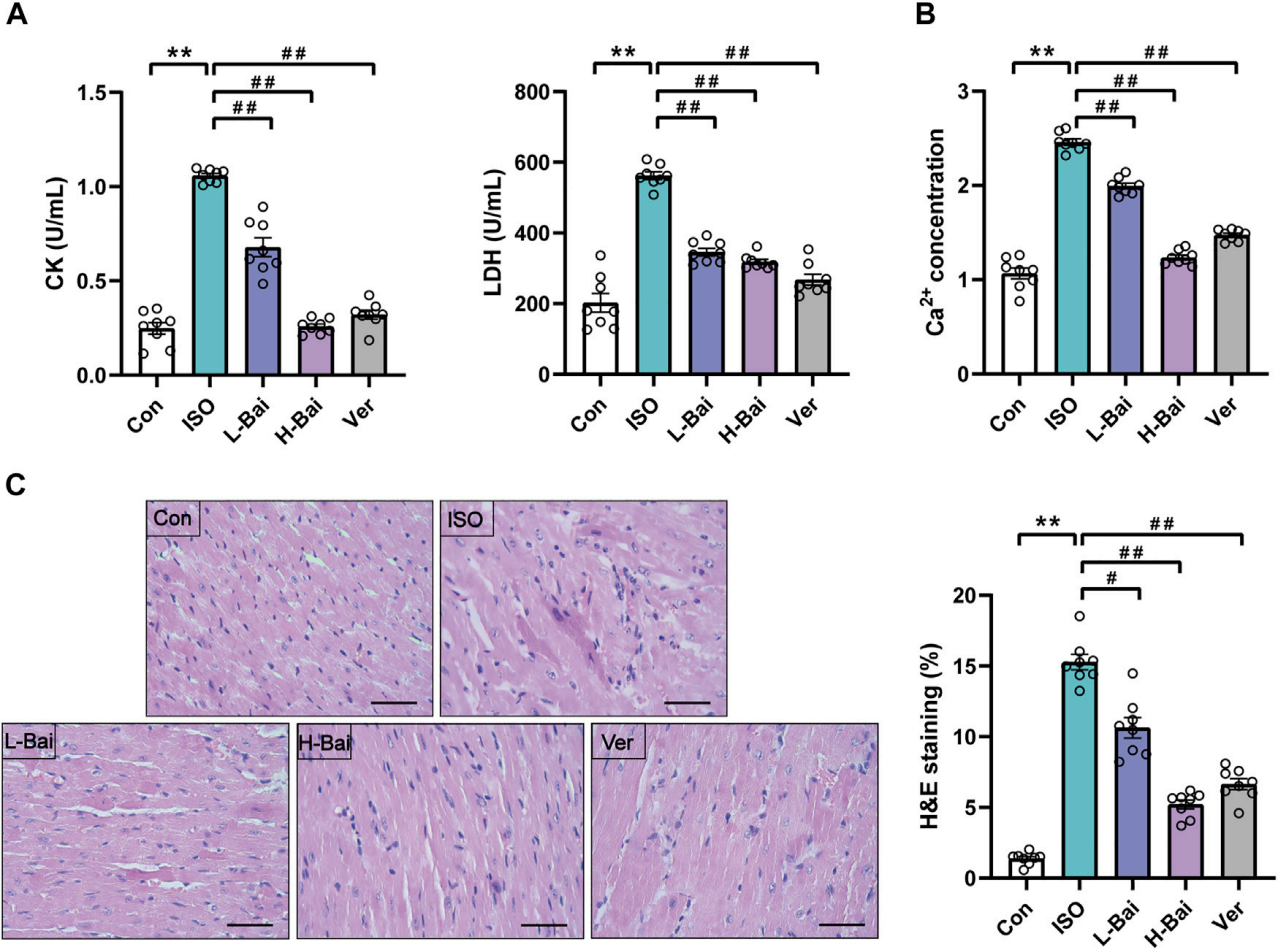
**(A)** Effects of Bai on levels of creatinine kinase and lactate dehydrogenase (CK and LDH, respectively) **(B)** concentration of Ca^2+^ in mice. **(C)** Representative heart histopathological images from Con, ISO, L-Bai, H-Bai, and verapamil (Ver) in mice, and the area of myocardial injury in each group was calculated. Scale bar = 50 μm (magnification: ×400).

As presented in Figures 3A,B, the mouse serum levels of CK, LDH, and myocardium Ca^2+^ concentrations in the ISO group were higher than those in the Con group (*p <* 0.01), while L-Bai, H-Bai, and Ver groups showed a significant recovery to varying degrees (*p <* 0.01).

### 3.4 Effects of Bai on Histopathology

Figure 3C presented the histopathology change of mouse hearts. Cardiac slices in the Con group showed a normal myofibrillar structure, while the ISO group demonstrated evident degeneration, infiltrating inflammatory cells, and necrosis. The necrosis and inflammatory cells in the Bai and Ver groups were less than those in ISO group. These changes in pathological structures were dramatically increased in the ISO group compared to the Con group (*p <* 0.01). Conversely, the L-Bai, H-Bai, and Ver groups reduced these pathological structural changes with increasing dose compared with the ISO group (*p <* 0.01 or *p <* 0.05).

### 3.5 Effects of Bai on Oxidative Stress

#### 3.5.1 Effects of Bai on ROS Generation

**FIGURE 4:**
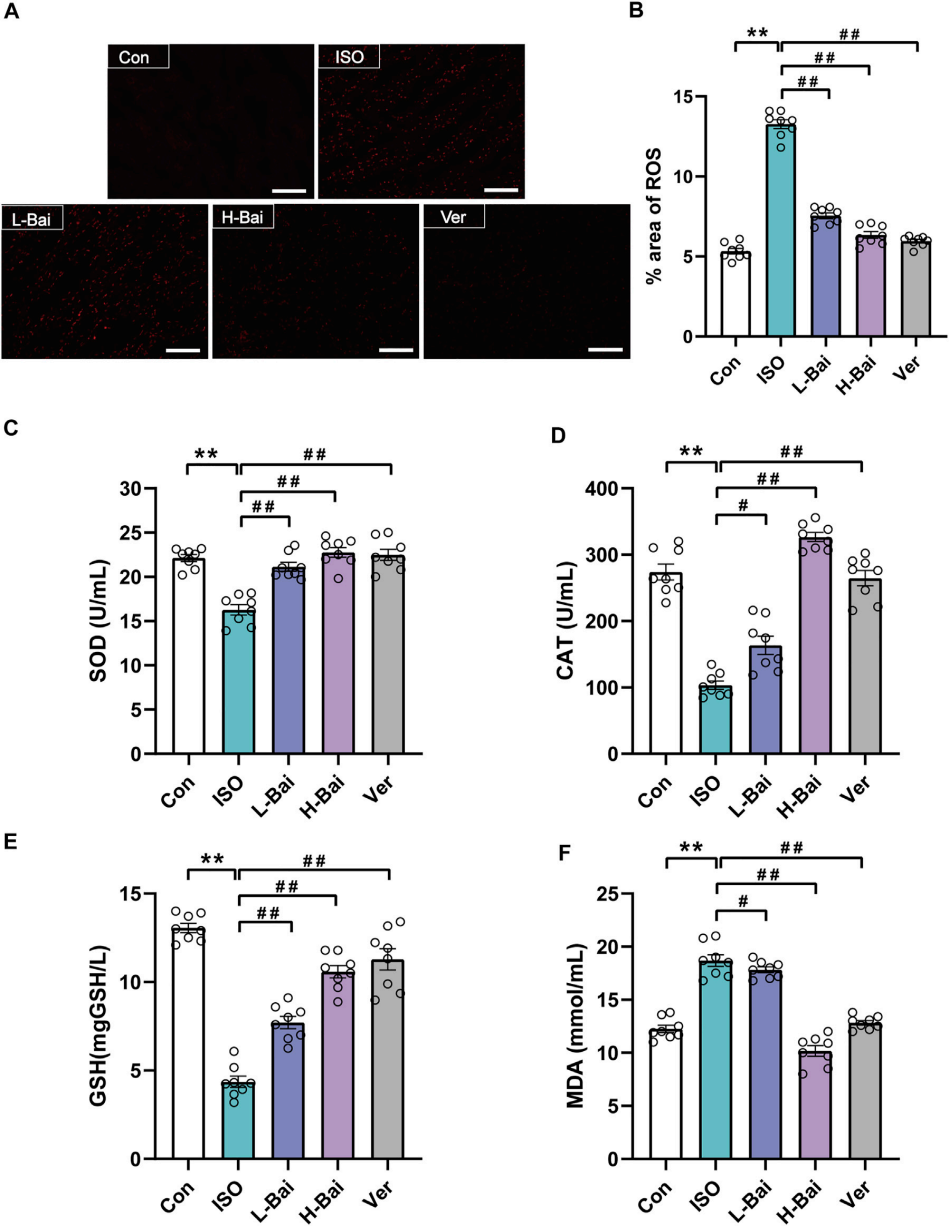
Effects of Bai on oxidative stress. **(A)** The fluorescence intensity images for reactive oxygen species (ROS) were observed by fluorescent probe dihydroethidium (DHE) at a magnification: ×200 in each group. Scale bar = 100 μm. **(B)** The area percentages of ROS levels were calculated. **(C-F)** Effects of Bai on activities of superoxide dismutase, catalase, and glutathione (SOD, CAT, GSH, respectively) and concentration of malondialdehyde (MDA). Values are denoted by means ± S.E.M. *p <* 0.01 versus Con; *p <* 0.01, *p <* 0.05 versus ISO.

The fluorescence of a dihydroethidium probe was applied to determine ROS generation in mouse cardiac tissue. As seen in Figures 4A,B, no obvious dichloro-fluorescein fluorescence was detected in the Con group. An abundance of ROS in the ISO group was detected. L-Bai, H-Bai, and Ver groups led to a significant reduction in ROS production compared to the ISO group. The results suggested that Bai decreased the ROS generation in a dose-dependent manner.

#### 3.5.2 Effects of Bai on SOD, CAT, GSH, and MDA

As shown in Figures 4C–F, the activities of SOD, CAT, and GSH of mouse serum in the ISO group significantly declined, while the level of MDA was noticeably elevated (*p <* 0.01). In contrast to the ISO group, pretreatment of L-Bai, H-Bai, and Ver caused an increase in SOD, CAT, and GSH activities and a reduction in MDA levels (*p <* 0.01 or *p <* 0.05). It was revealed that Bai reduced the levels of oxidative stress in a dose-dependent manner.

### 3.6 Effects of Bai on Inflammatory Markers of IL-6 and TNF-α

**FIGURE 5:**
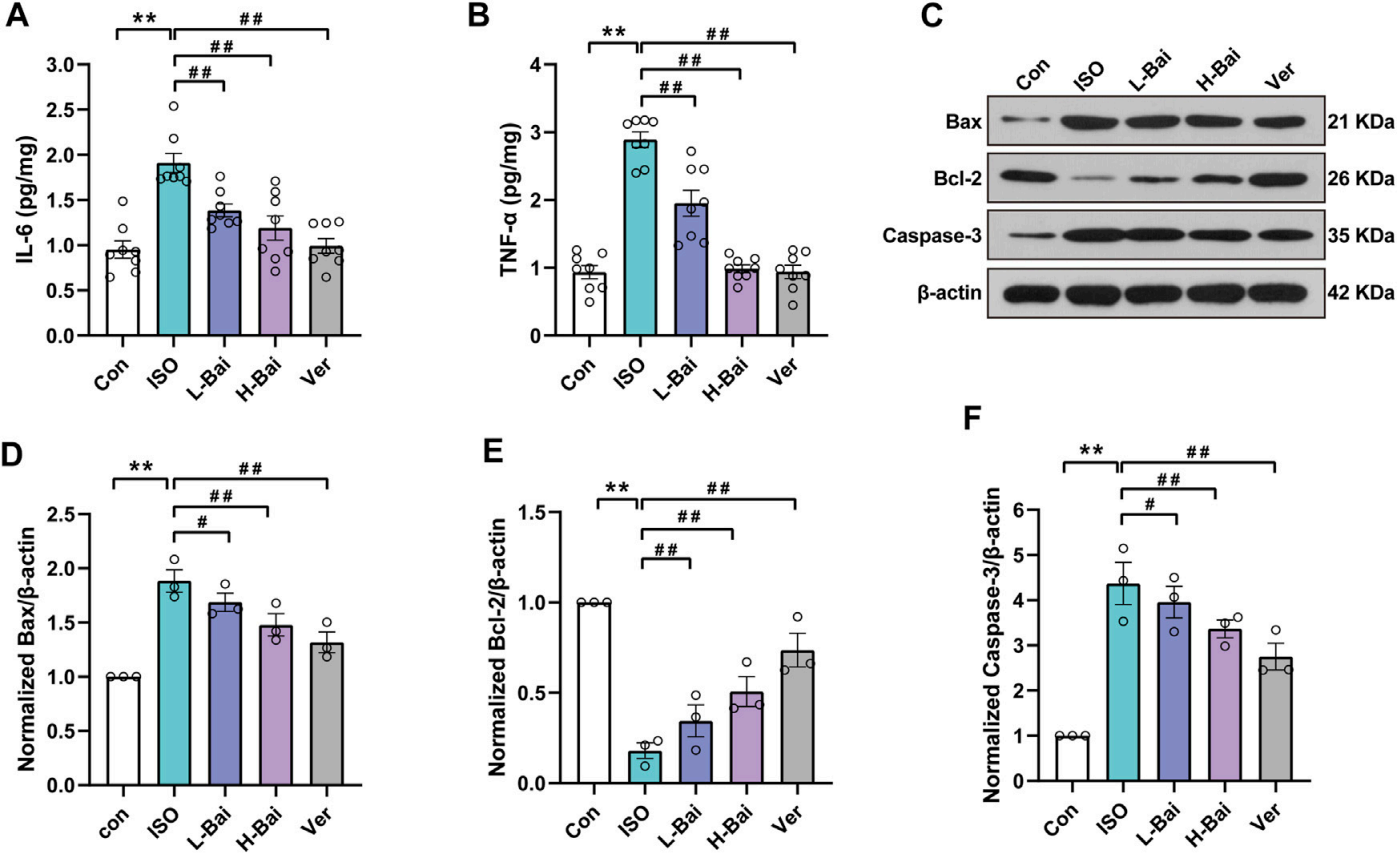
Effects of Bai on interleukin 6, tumour necrosis factor alpha (IL-6, TNF-α), Bcl-associated X protein, B-cell lymphoma 2 (Bax and Bcl-2, respectively) and Caspase-3 in mouse myocardium. **(A,B)** IL-6 and TNF-α levels were measured by enzyme-linked immunosorbent assay (ELISA). **(C)** Western blotting was applied to measure the protein expressions of Bax, Bcl-2, and Caspase-3. **(D-F)** Relative intensities of Bax, Bcl-2, and Caspase-3 were normalized to β-actin. Values are presented as means ± S.E.M. *p <* 0.01 versus Con; *p <* 0.01, *p <* 0.05 versus ISO.

In contrast with the Con group, the results indicate that IL-6 and TNF-α in the ISO treated group were significantly elevated (Figures 5A,B, *p <* 0.01). Meanwhile, the L-Bai, H-Bai, and Ver groups were found to cause a decrease in the levels of IL-6 and TNF-α to varying degrees in mice (*p <* 0.01 or *p <* 0.05). Our findings suggested that Bai inhibited the levels of inflammatory factors in a dose-dependent manner.

### 3.7 Effects of Bai on Apoptosis Markers of Bax, Bcl-2, and Caspase-3

We determined protein levels of the apoptotic markers based on Western blots to observe the expression of Bax, Bcl-2, and Caspase-3 factors that are mainly associated with apoptosis (Figures 5C–F). Compared to the Con group, the ISO group showed a noticeable increase (*p <* 0.01), while the Bcl-2 level showed a significant decrease. Mice given L-Bai, H-Bai, and Ver showed a reduction in Bax and Caspase-3 levels (*p <* 0.01 or *p <* 0.05), while the Bcl-2 level showed enhancement during the same period compared to the ISO group (*p <* 0.01 or *p <* 0.05). It was manifested that Bai inhibited the expressions of apoptotic factors in a dose-dependent manner.

### 3.8 Effects of Bai on TLR4/MyD88/MAPKs/NF-κB Pathway

**FIGURE 6:**
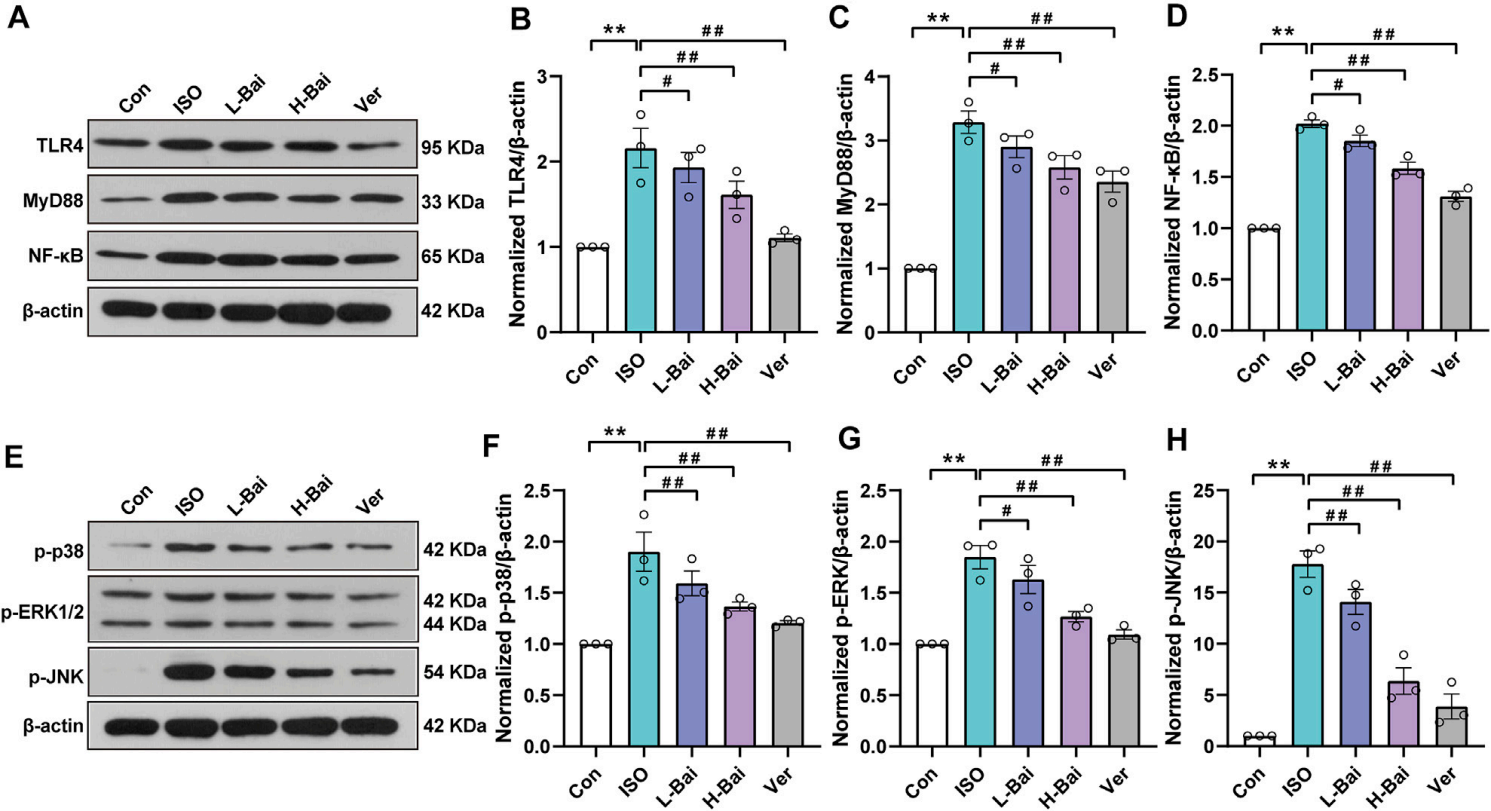
Effects of Bai on TLR4, MyD88, NF-κB, and MAPKs pathways. **(A,E)** Western blot was applied to measure the protein expressions of TLR4, MyD88, NF-κB, p-p38, *p*-ERK1/2, and *p*-JNK in mice. **(B-D)** Relative intensities of TLR4, MyD88, and NF-κB were normalized to β-actin **(F–H)** in addition to p-p38, p-extracellular-regulated kinase 1/2 (*p*-ERK1/2), and *p*-jun kinase (*p*-JNK). Values are presented as means ± S.E.M. *p <* 0.01 versus Con; *p <* 0.01, *p <* 0.05 versus ISO.

Figure 6A–H illustrates that the protein expressions of TLR4, MyD88, NF-κB, p-p38, *p*-ERK1/2, and *p*-JNK in ISO were significantly increased compared to the Con group (*p <* 0.01). In groups that mice were treated with L-Bai, H-Bai, and Ver, these protein expressions were significantly depressed (*p <* 0.01 or *p <* 0.05). The results revealed that Bai inhibited the expressions of TLR4/MyD88/MAPKs/NF-κB pathway in a dose-dependent manner.

### 3.9 Reduction of L-type Ca2+ Currents, Myocyte Contractions and Ca2+ Transients by Bai

#### 3.9.1 Confirmation of L-type Ca2+ Currents

**FIGURE 7:**
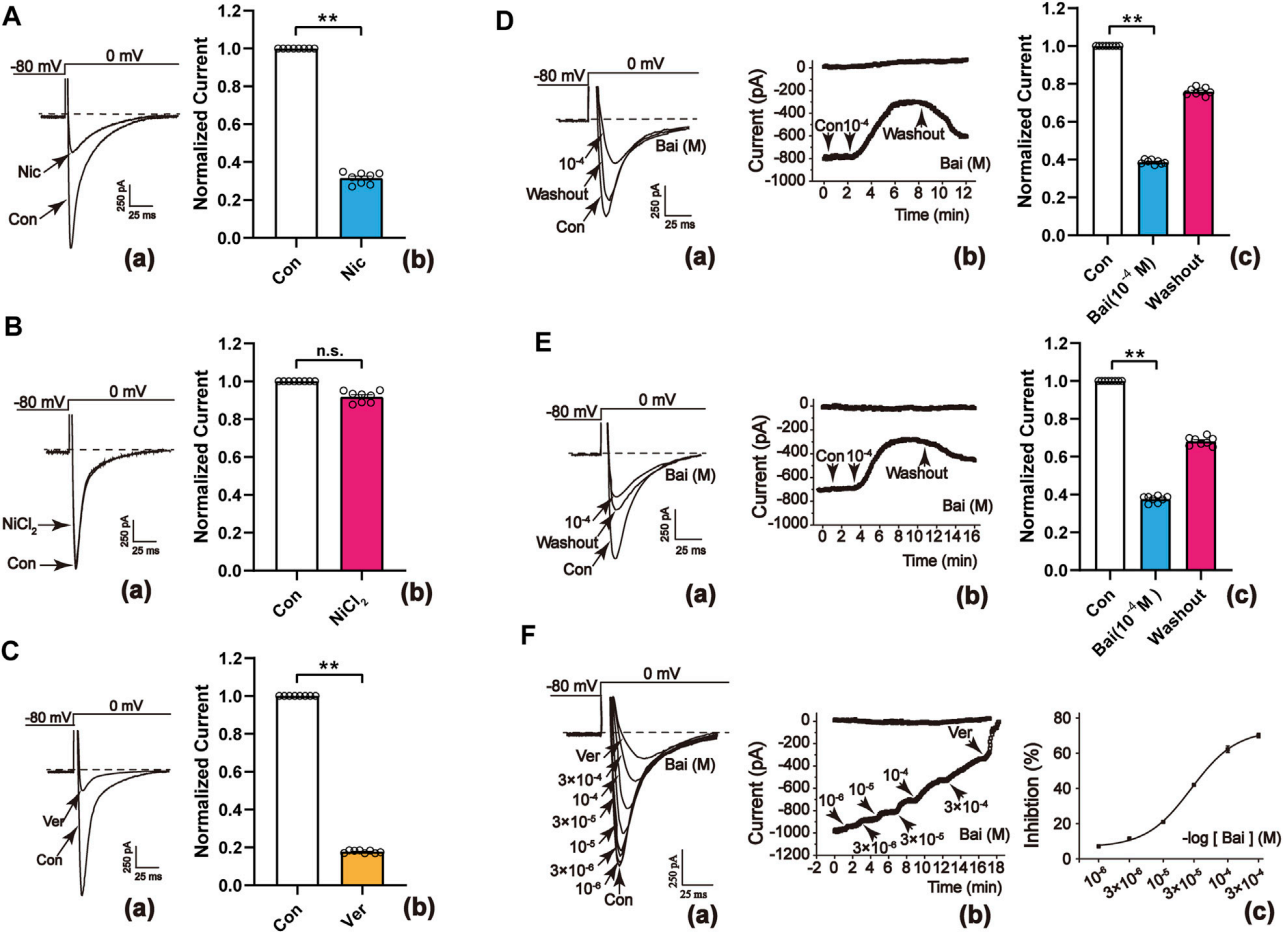
Confirmation of L-type Ca^2+^ currents in rat cardiomyocytes. **(A–C[a])** Typical traces and **(A–C[b])** summary data exhibited the representative L-type Ca^2+^ currents recordings with the application of **(A)** Nic (10^−3^ M) **(B)** NiCl_2_ (10^−2^ M), and **(C)** Ver (10^−6^ M). Nic and Ver almost totally blocked the L-type Ca^2+^ currents. Effects of Bai on L-type Ca^2+^ currents in **(D)** normal ventricular myocytes and **(E)** ischemic ventricular myocytes. **(D,E[a])** Typical traces **(D,E[b])** temporal course of L-type Ca^2+^ currents, and **(D,E[c])** summary data were recorded in the control conditions, during applications of Bai (10^−4^ M) and washout. **(F** **[a])** Current traces and **(F** **[b])** the temporal course of L-type Ca^2+^ currents were recorded in the control conditions during exposure to 10^−6^, 3 × 10^−6^, 10^−5^, 3 × 10^−5^, 10^−4^, and 3 × 10^−4^ M Bai, and 10^−6^ M Ver. **(F** **[c])** Concentration-response curve representing the percent inhibition of Bai. Values are expressed as means ± S.E.M. *p <* 0.01 versus Con, n = 6-8 cells.

L-type Ca^2+^ currents are determined using a steady-state activation protocol. The currents were almost totally blocked by Nic (10^−3^ M), a specific LTCC antagonist, indicating that they were LTCC (*p* < 0.01) as shown in Figures 7Aa,b. As a specific T-type Ca^2+^ channel blocker, NiCl_2_ (10^−2^ M) did not affect the currents (Figure 7Ba,b), indicating that no existence of T-type Ca^2+^ currents could be found. Ver (10^−6^ M) is a specific L-type Ca^2+^ currents inhibitor and capable of almost completely inhibiting current flow (*p <* 0.01, Figure 7Ca,b). These findings indicated that the currents in rat cardiomyocytes were evoked by LTCC.

#### 3.9.2 Inhibitory Effects of Bai on L-type Ca2+ Currents in Normal and Ischemic Ventricular Myocardial Cells

Figure 7D, E show that the peak of L-type Ca^2+^ currents was significantly inhibited (*p <* 0.01) by 61.57 ± 1.03% and 60.26 ± 1.42% after administration of 10^−4^ M Bai. Nevertheless, L-type Ca^2+^ currents partially recovered after being subject to washout with an external solution. The results demonstrated that Bai inhibits L-type Ca^2+^ currents in normal and ischemic rat cardiomyocytes, which appears to be a partially reversible process.

#### 3.9.3 Concentration-dependent Effects of Bai on L-type Ca2+ Currents

Figure 7Fa exhibits the representative current traces evoked from the test potentials of –80 to 0 mV at different Bai concentrations. The time course of the peak L-type Ca^2+^ currents were continuously reduced after using increasing concentrations of Bai (10^−6^, 3 × 10^−6^, 10^−5^, 3 × 10^−5^, 10^−4^, 3 × 10^−4^ M) or Ver (10^−6^ M) as shown in Figure 7Fb. The half-maximal inhibitory concentration (IC_50_) of Bai was 4.7295 × 10^−5^ M. The inhibition rates by Bai at 10^−6^, 3 × 10^−6^, 10^−5^, 3 × 10^−5^, 10^−4^, and 3 × 10^−4^ M were 7.97 ± 0.15%, 12.95 ± 0.85%, 21.5 ± 0.8%, 45.45 ± 0.6%, 61.57 ± 1.03%, and 76.67 ± 0.5%, respectively (Figure 7Fc).

#### 3.9.4 Effects of Bai on the Current–Voltage (I–V) Relationship of L-type Ca2+ Currents

**FIGURE 8:**
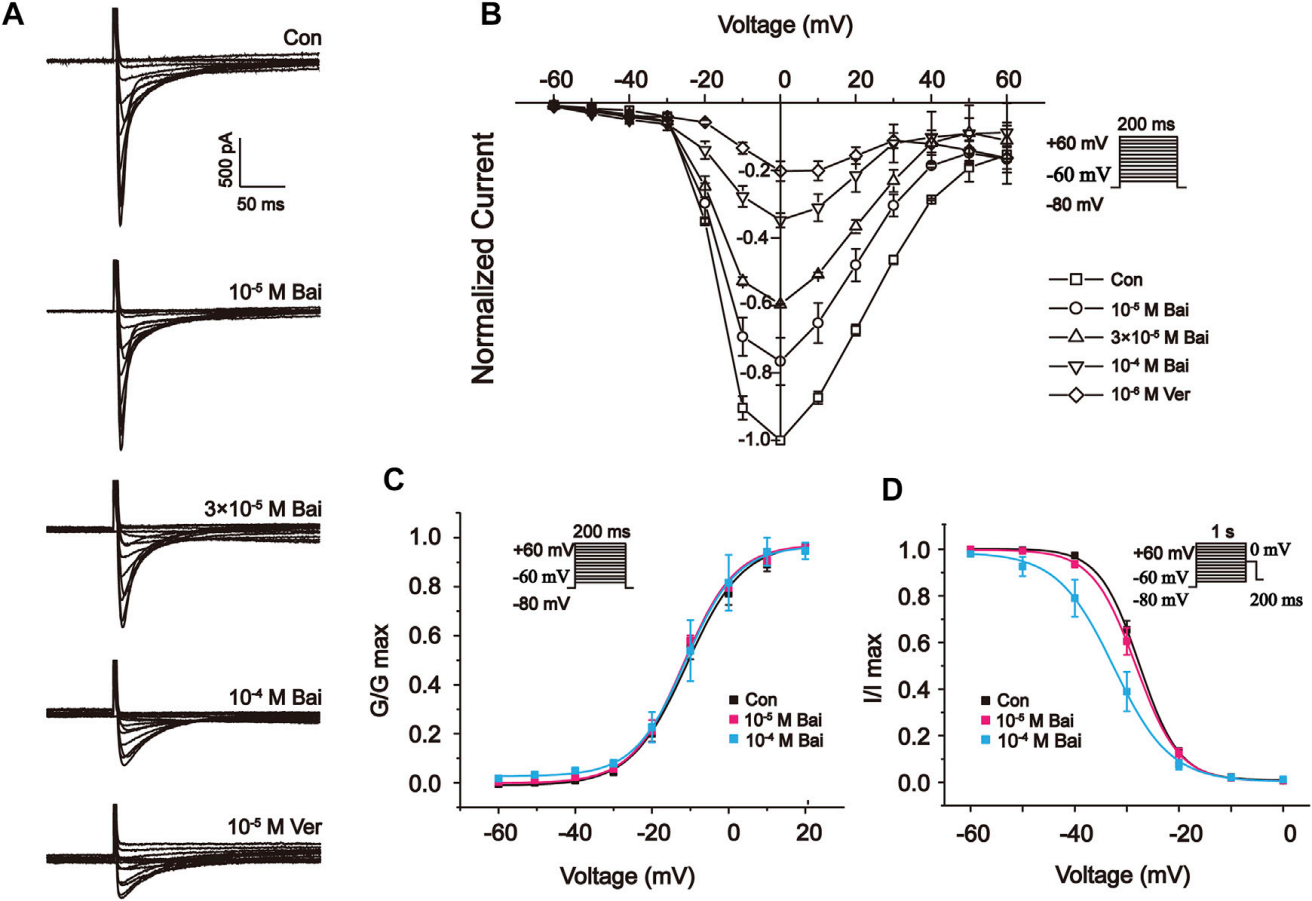
Effects of Bai on the I-V relationship of L-type Ca^2+^ currents in rat cardiomyocytes. **(A)** Typical traces and **(B)** summary data are exhibited under the application of Con (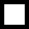), Bai at 10^−5^ M (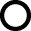), 3 × 10^−5^ M (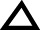), 10^−4^ M (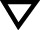), and Ver at 10^−6^ M (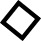). Effects of Bai on steady-state activation and inactivation of L-type Ca^2+^ currents. **(C)** Activation curves and **(D)** inactivation curves of L-type Ca^2+^ currents exhibited under the application of Con and Bai at 10^−5^ M and 10^−4^ M. Values are presented as means ± S.E.M. n = 6-8 cells.

Figure 8A,B show the I–V relationship curves at distinct concentrations of Bai (10^−5^ M, 3 × 10^−5^ M, and 10^−4^ M) and Ver (10^−6^ M). The currents appeared between –60 and 60 mV. Also, Bai produced a dose-dependent effect on I–V curves. The amplitudes of L-type Ca^2+^ currents were enhanced at –20 mV. Nevertheless, the I–V relationship of L-type Ca^2+^ currents and the reversal potential did not significantly change after these treatments.

#### 3.9.5 Effects of Bai on Steady-State Activation and Inactivation of L-type Ca2+ Currents

The voltage-dependent effects of different Con and Bai concentrations (10^−5^ and 10^−4^ M) on the state of steady activation and inactivation of L-type Ca^2+^ currents were shown in Figures 8C,D. However, Bai generated an obvious leftward shift of the inactivation curve of L-type Ca^2+^ currents. The values of V_1/2_ and slope factor (k) for the activation of Con and Bai (10^−5^ and 10^−4^ M) were –10.94 ± 1.05 mV/7.277 ± 0.95, –11.78 ± 0.89 mV/6.79 ± 0.80, and –11.24 ± 0.41 mV/6.69 ± 0.37, respectively. The values of V_1/2_ and slope factor (k) for inactivation in the Con and Bai (10^−5^ and 10^−4^ M) groups were –27.91 ± 0.75 mV/3.7979 ± 0.75, –28.62 ± 0.76 mV/4.10 ± 0.77, and –32.51 ± 0.52 mV/5.41 ± 0.47, respectively.

#### 3.9.6 Effects of Bai on Myocyte Contractions and Ca2+ Transients

**FIGURE 9:**
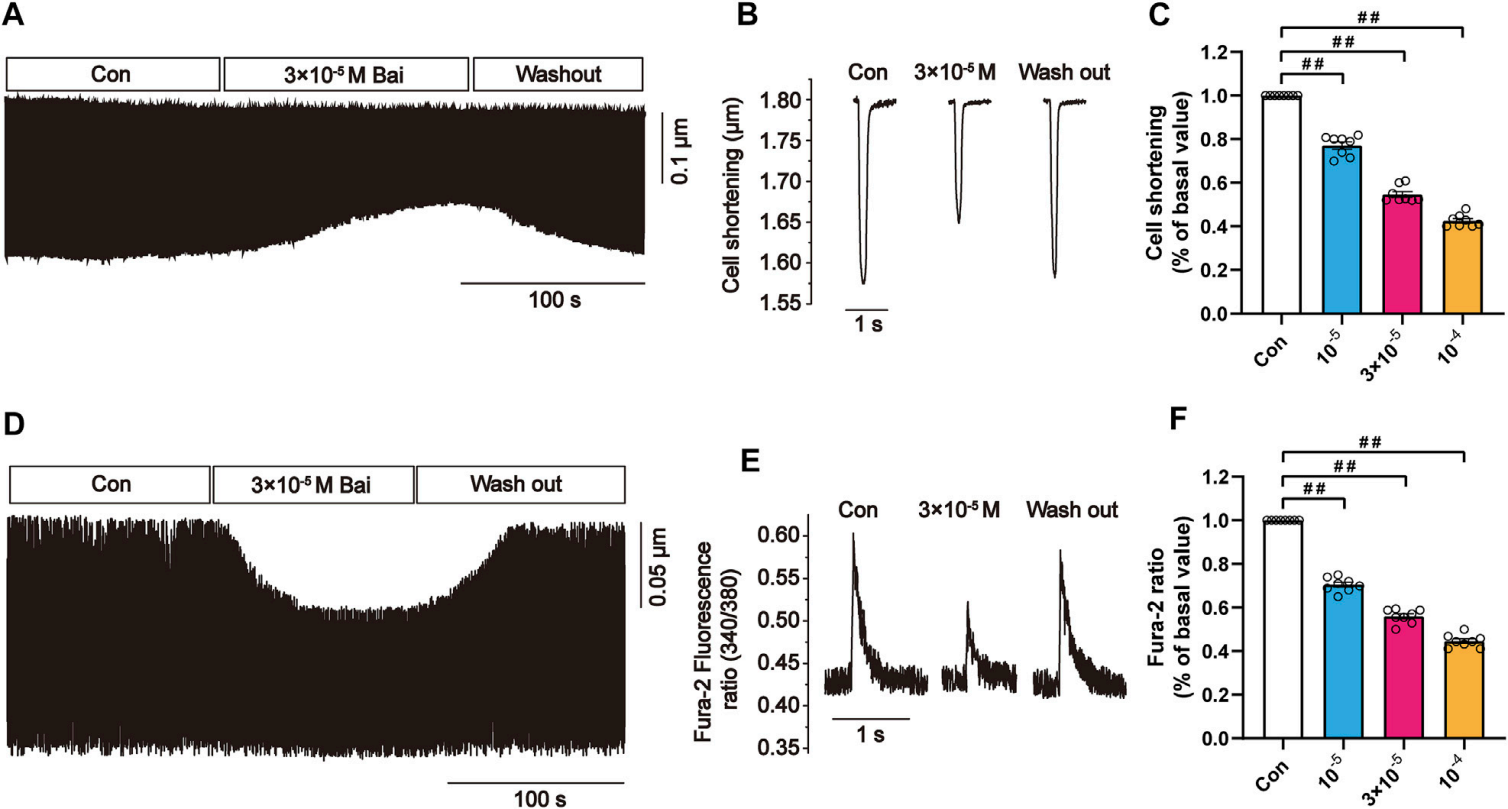
Effects of Bai on myocyte contractions and Ca^2+^ transients in rat cardiomyocytes. **(A,D)** Time course and **(B,E)** typical traces recorded in Con and Bai (3 × 10^−5^ M). **(C,F)** Pooled data of myocyte contractions and Ca^2+^ transients under Con and Bai (10^−5^, 3 × 10^−5^, and 10^−4^ M). Values are presented as means ± S.E.M. *p <* 0.01, *p <* 0.05 versus Con, n = 6-8 cells.

Changes in rat myocyte contractions in Con and Bai groups are shown in Figure 9A,B in which the representative myocyte contractions in the absence and presence of Bai (3 × 10^−5^ M) are displayed. It can be seen that contractility after wash-out was partially recovered. The results show that Bai significantly inhibited myocyte shortening by 39.19 ± 0.84%, 51.495 ± 1.905%, and 61.96 ± 3.92% (*p <* 0.01) at doses of 10^−5^, 3 × 10^−5^, and 10^−4^ M, respectively (Figure 9C). Figures 9D–F shows the variations in Ca^2+^ transient with/without Bai (10^−5^, 3 × 10^−5^, 10^−4^ M). The representative Ca^2+^ transients in both the presence and absence of Bai (3 × 10^−5^ M) are displayed in Figures 7D,E. The amplitudes of Ca^2+^ transients decreased by 30.57 ± 1.19%, 42.64 ± 1.36%, and 59.2 ± 2.18% at concentrations of 10^−5^, 3 × 10^−5^, and 10^−4^ M, respectively (*p <* 0.01 or *p <* 0.05) as shown in Figure 7F.

### 3.10 Effects of Bai on Cultured H9c2 Cardiomyocytes

#### 3.10.1 Effects of Bai on H9c2 Cells Inflammatory Markers of IL-6 and TNF-α

**FIGURE 10:**
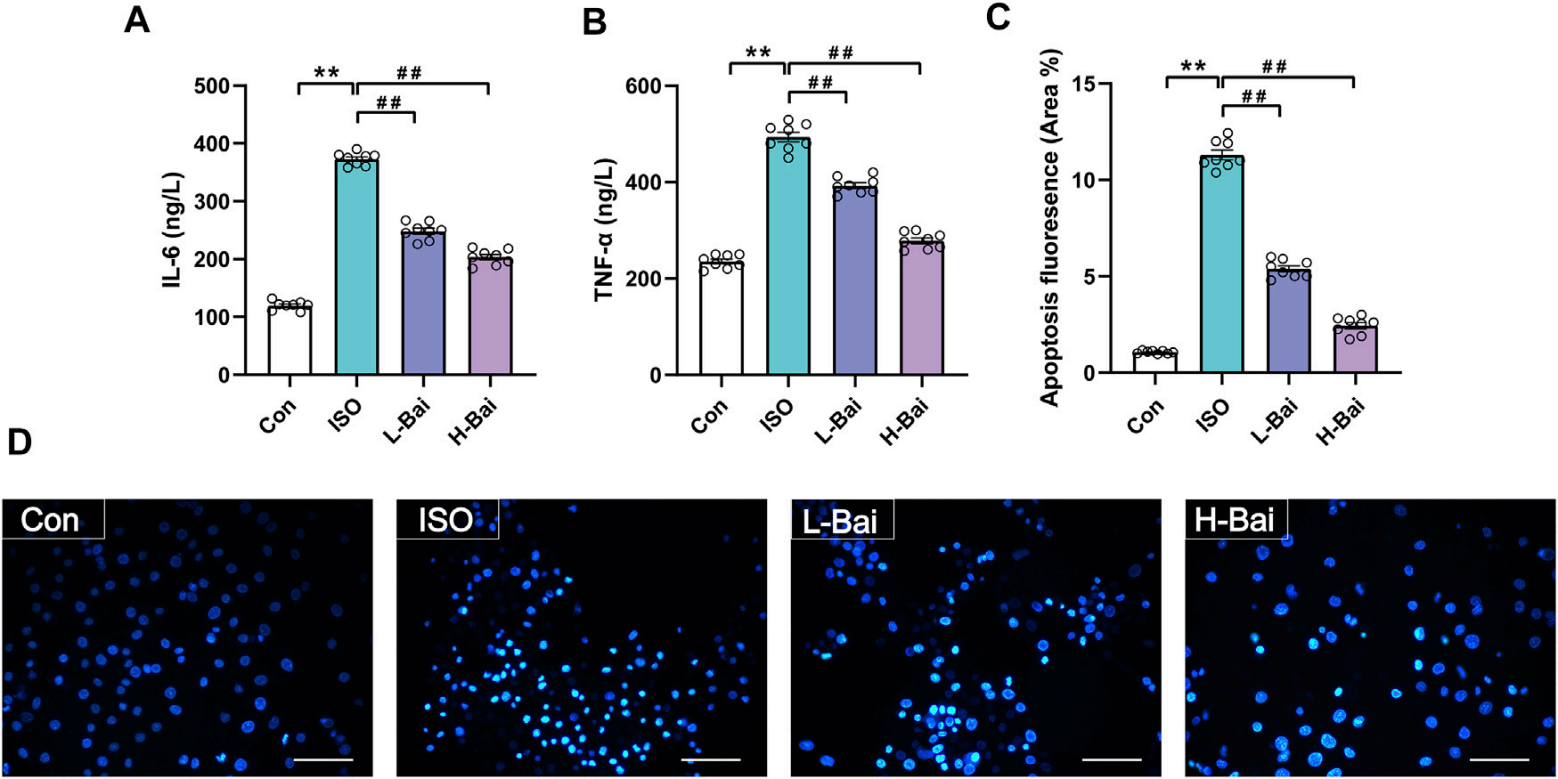
**(A,B)** Effects of Bai on levels of IL-6 and TNF-α in H9c2 cells. **(C,D)** Effects of Bai on apoptosis of MI cardiomyocytes by Hochest 33258 staining. Apoptotic fluorescence pictures from different groups; and the ratio diagram about fluorescence intensity of apoptosis is exhibited. Values are presented as means ± S.E.M. *p <* 0.01 versus Con; *p <* 0.01 versus ISO group, n = 6.

As shown in Figures 10A,B the levels of IL-6 and TNF-α in cultured H9c2 cardiomyocytes were significantly upregulated in ISO groups (*p <* 0.01); However, these levels significantly decreased after Bai administration (*p <* 0.01 or *p <* 0.05). The results indicate that Bai could ameliorate ISO-induced inflammatory injury.

#### 3.10.2 Effects of Bai on H9c2 Cells Apoptosis by Hoechst 33258 Staining

Myocardial apoptosis was demonstrated as the presence of areas of Hoechst 33258 staining (Figures 10C,D). The intensity of fluorescence from the ISO group significantly increased when compared with Con group (*p <* 0.01). Fluorescence was significantly downregulated in the L-Bai and H-Bai groups (*p <* 0.01). The results indicate that Bai exerts protective effects against ISO-induced myocardial apoptosis.

## 4 Discussion

As the leading cause of fatality worldwide, MI has become a serious threat to human health (Priori et al., 2015). At present, the medications used to treat MI still have many adverse effects, so it is particularly important to find other alternative drugs with minimal side effects.

It is an effective approach to establish animal models for investigating the prevention and treatment of ischemic heart disease, particularly MI. An experimentally ISO-induced MI model could show changes in the ECG, cardiac shape, and marker enzymes similar to human MI (Karthick and Stanely Mainzen Prince, 2006). MI signs on an ECG are ST-segment elevation and heart rate acceleration. In our study, heart coefficients were reduced after exposure to Bai. Also, a previous study demonstrated that ISO causes myocardial cell damage and loss of cell membrane function integrity, which stimulates the release of cardiac marker enzymes into the blood (Constant, 1997). As shown in Figure 3A, Bai could cause a reduction in the levels of serum CK and LDH in mice. Besides, Ca^2+^ overload is closely associated with MI (Zhang et al., 2018), so we measured Ca^2+^ concentrations in heart tissues. The results indicated that Bai treatment inhibited Ca^2+^ influx. Also, the significant improvement in histopathological examination in mice further demonstrated the cardioprotective effect of Bai.

ROS is a critical element in the pathogenesis of ISO-inducing MI (Patel et al., 2010). The autoxidation of high dose ISO produces highly toxic ROS that disrupts the balance between pro and antioxidants and enhances lipid peroxidation. In addition, antioxidant defense systems composed of various antioxidant enzymes and antioxidants exist within cells. Our results show that Bai could lead to a decrease in the production of ROS; moreover, Bai caused a significant enhancement of SOD, CAT, and GSH activities and a reduction in MDA levels in mice (Figure 4), which were consistent with previously described studies (Haleagrahara et al., 2011; Han et al., 2019). The antioxidant capacity of Bai appears to be the potential mechanism against ISO-induced MI.

TLR4 and NF-κB signaling pathways could be activated by excessive ROS (X. Wang et al., 1998). The TLR4 signaling pathway is an important mediator of myocardial injury induced by inflammation (Jenke et al., 2013). Activation of TLR4 leads to an improvement in the MyD88 level, which is one of the major adaptor proteins for TLR4 involved in promoting signal transduction (Medzhitov et al., 1998). In the meantime, the MyD88-dependent pathway might activate MAPKs and the NF-κB pathway (Keating et al., 2007; G.; Wang et al., 2017). Results in our study indicated that injection of ISO caused an increase in pro-inflammatory cytokine levels, such as IL-6 and TNF-α, via activation of the TLR4 and NF-κB signaling pathways. Bai could also cause a decrease in ISO-induced inflammation in H9c2 cardiomyocytes as shown in Figure 10. These factors decreased after pretreatment with Bai, indicating that Bai could attenuate cardiac injury by inhibiting inflammatory reactions. Therefore, we speculate that the protective effects of Bai on heart injury might be related to inhibition of the signaling described above.

NF-κB can activate MAPKs, and MAPKs regulate anti-apoptotic proteins (W. Gao et al., 2016). MAPKs, a family of signaling proteins, are chiefly activated as a response to extracellular stimulation (Ghasemi et al., 2014) and act as a signal amplifier to coordinate cellular responses, such as apoptosis (Cao et al., 2014). Three members of this family have been found to date: 1) ERK, 2) JNK, and 3) p38. ERK is regarded as an anti-apoptotic factor. The activated form of *p*-ERK can promote cell survival, proliferation, and differentiation (Maemura et al., 2009). JNK and p38 are considered proapoptotic factors. JNK is one of the most significant subfamilies in the MAPK system, and activated JNK (*p*-JNK) can lead to apoptosis (Kamata et al., 2005). The p38 plays a vital role in inflammatory response and apoptosis; its activation is regarded as an early requirement for apoptosis (Galán et al., 2000; Fang et al., 2019). The reduction in ERK1/2, p38, and JNK expression demonstrates that Bai could inhibit the MAPK pathway. ROS can activate the caspase family proteases to generate apoptosis (Kluck et al., 1997). Cardiomyocyte apoptosis is considered to be a critical event of ischemic heart disease (Eefting et al., 2004; Logue et al., 2005). Our study shows that Bai caused an increase in the anti-apoptotic Bcl-2 expression and a reduction in the expression of pro-apoptotic Bax and Caspase-3 in mouse myocardium. These results might be related to the suppression of the MAPK signaling pathway. In addition, the results of apoptotic fluorescence showed that Bai could cause a decrease in the ISO-induced apoptosis of H9c2 cardiomyocytes (Figure 10).

The crucial pathogenic feature of MI is abnormal myocardial Ca^2+^ homeostasis (Madamanchi and Runge, 2013). The separated myocyte models provide a chance to inspect the physiological adaptations in heart function. Calcium, an omnipresent indicator, is involved in an extensive range of cell activities (Berridge et al., 2003; Clapham, 2007). Ca^2+^ induces excitation-contraction coupling, and membrane depolarization evokes voltage-gated Ca^2+^ channels, enables Ca^2+^ to enter cardiomyocytes through LTCC, and then triggers the sarcoplasmic reticulum to release Ca^2+^, which increases intracellular Ca^2+^. Nevertheless, the increase in mechanical contraction in myocardial cells was induced by Ca^2+^ overload. Moreover, Ca^2+^ storage contributes to the generation of Ca^2+^ transients. Therefore, the inhibition of LTCC could reduce L-type Ca^2+^ currents, myocardial contractility, and Ca^2+^ transients.

Our data show that Bai produced a dose-dependent decrease in L-type Ca^2+^ currents in rat cardiomyocytes. Bai at 3 × 10^−4^ M caused a decrease in L-type Ca^2+^ currents in ischemic rat ventricular myocytes. Bai does not cause a change in the I–V relationship and the L-type Ca^2+^ current reversal potential. Also, the L-type Ca^2+^ currents inactivation curves are shifted by Bai (10^−5^, 10^−4^ M) in the negative direction, which slows the recovery due to inactivation. Accordingly, we assume that activation and inactivation might be mechanisms by which Bai inhibits L-type Ca^2+^ currents. Furthermore, Bai was shown to inhibit contractility and Ca^2+^ transients (Figure 9). Ischemia led to induction of membrane depolarization and an increase in intracellular Ca^2+^ influx. The increase in intracellular Ca^2+^ expedites the activities of some enzymes that consume ATP, which further depletes energy stores and makes the cardiac tissue more vulnerable to ischemia injury (Maltsev et al., 1998). Our data show that Bai can cause inhibition in the increase in intracellular Ca^2+^ by inhibiting LTCC and leading to a reduction in extracellular Ca^2+^ influx. Ca^2+^ influx is required for excitation-contraction coupling in all cardiomyocytes. Therefore, the inhibitory effects of Bai on the contractility might be achieved via a reduction in Ca^2+^ influx. The reduction in contractility is another mechanism by which Bai may reduce the oxygen requirement in MI animals. In conclusion, these results demonstrate the cardioprotective effects and underlying protective mechanisms of Bai on ventricular myocytes.

Some limitations in the present research should be considered. Myocardial oxygen consumption was not directly examined, which is of great significance when evaluating anti-ischemic drugs. A heart rate increase at rest is intimately associated with acute and chronic heart failure (Oliva et al., 2018); tachycardia is also the cause of acute MI (Sahyoun and Hicks, 1981; Heusch, 2008), so the reduction of heart rate is critical in the relief of acute MI and heart failure (Ferrari and Fox, 2016). However, it is not clear whether Bai can lead to a reduction in the ISO-induced acceleration of heart rate and could be considered a mechanism of myocardial protection in our present experiment. These limitations in our current experiment need to be addressed in future studies.

## 5 Conclusion

In summary, this study showed that Bai exhibited cardioprotective effects on ISO-induced MI. The potential mechanism might be associated with the regulation of oxidative stress, inflammatory response, and apoptosis. This process might be achieved via suppression of the TLR4/MyD88/MAPK_S_/NF-κB pathways. In addition, inhibition of LTCC and dampening of intracellular Ca^2+^ may also contribute to alleviating MI injury. In brief, Bai can not only play a vital function in heart disease through antioxidant-, anti-inflammatory-, and apoptosis-related activities but also by protecting the heart by inhibiting LTCC. This study can serve as a basis for future research. Bai is regarded as an LTCC inhibitor, which provides a new perspective for the treatment of cardiovascular diseases but demands further exploration.

## Data Availability

The original contributions presented in the study are included in the article/Supplementary Material, further inquiries can be directed to the corresponding authors.

## Abbreviation

Bai, baicalein; Bax, Bcl-2-associated X; Bcl-2, B cell lymphoma-2; CAT, catalase; CK, creatinine kinase; Con, control; ECG, electrocardiogram; GSH, glutathione; ICa-L, L-type Ca2+ currents; IL-6, interleukin-6; ISO, isoproterenol; L-Bai, Low-dose Bai; LDH, lactate dehydrogenase; LTCC, L-type Ca2+ channels; MAPKs, mitogen-activated protein kinases; MDA, malondialdehyde; MI, myocardial ischemia; MyD88, myeloid differentiation protein 88; Nic, nicardipine; NF-κB, nuclear factor-kappa B; *p*-ERK1/2, p-extracellular signal-regulated kinase1/2; *p*-JNK, c-Jun N-terminal kinase; ROS, reactive oxygen species; SOD, superoxide dismutase; TNF-α, tumor necrosis alpha; TLR4, Toll-like receptor-4; Ver, verapamil.
