# Supplementary figures and images for: Baicalein Ameliorates Myocardial Ischemia Through Reduction of Oxidative Stress, Inflammation and Apoptosis via TLR4/MyD88/MAPKS/NF-κB Pathway and Regulation of Ca2+ Homeostasis by L-type Ca2+ Channels
Source: Front Pharmacol. 2022 Mar 16;13:842723. doi: 10.3389/fphar.2022.842723 (PMC8967179; doi:10.3389/fphar.2022.842723)

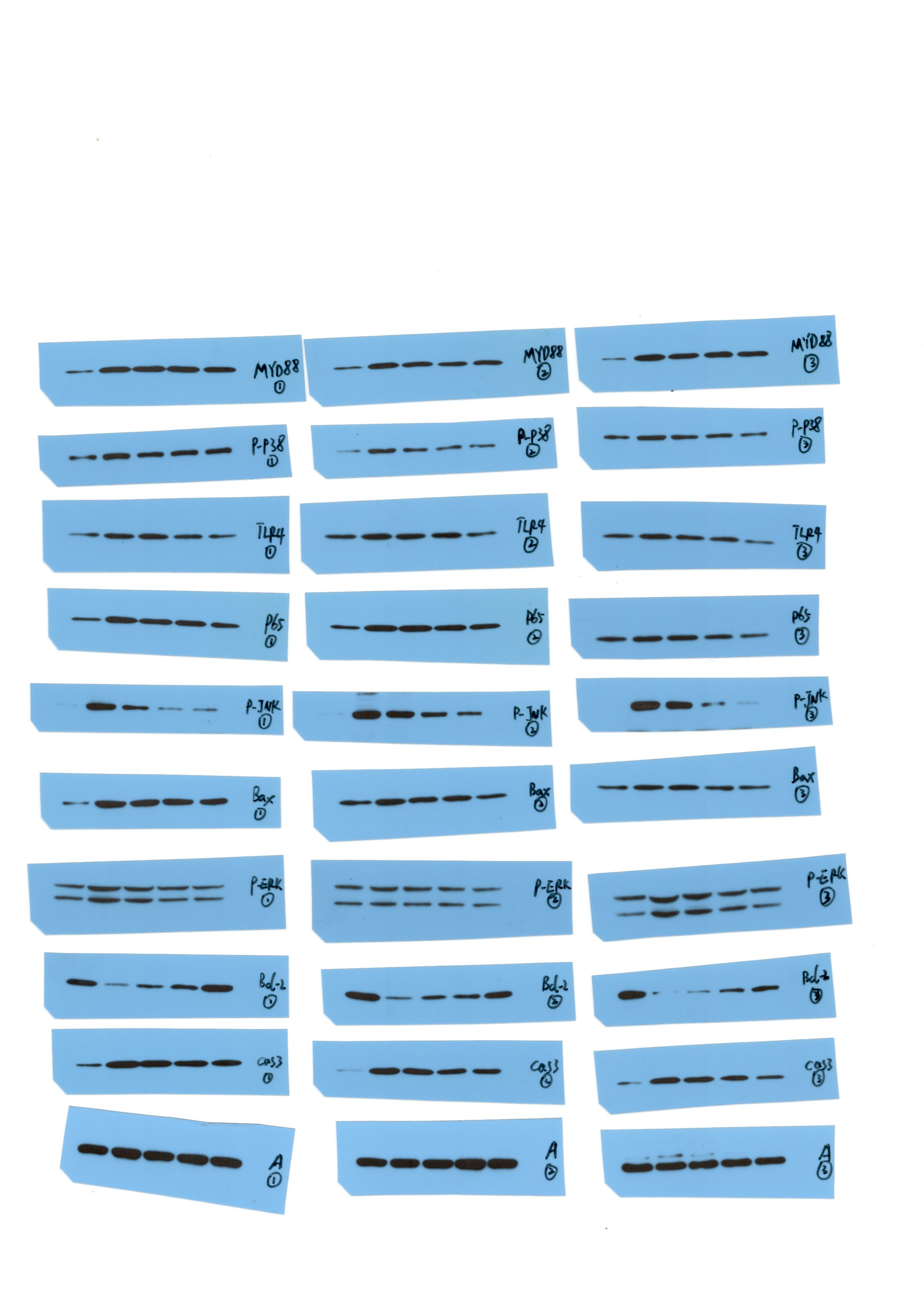

Supplement: Supplementary file 1 [file Image1.TIF]
